# Protein Marker-Dependent Drug Discovery Targeting Breast Cancer Stem Cells

**DOI:** 10.3390/ijms26167935

**Published:** 2025-08-17

**Authors:** Ashley V. Huang, Yali Kong, Kan Wang, Milton L. Brown, David Mu

**Affiliations:** 1 Department of Biomedical and Translational Sciences, Macon & Joan Brock Virginia Health Sciences at Old Dominion University, Norfolk, VA 23507, USA; huangav@odu.edu (A.V.H.); kongy@odu.edu (Y.K.); wangk@odu.edu (K.W.); 2 Department of Internal Medicine, Macon & Joan Brock Virginia Health Sciences at Old Dominion University, Norfolk, VA 23507, USA; brownml@odu.edu; 3 Leroy T. Canoles Jr. Cancer Research Center, Macon & Joan Brock Virginia Health Sciences at Old Dominion University, Norfolk, VA 23507, USA

**Keywords:** breast cancer stem cell, DCLK-1, ALDH1, circadian rhythm, infradian rhythms, drug discovery

## Abstract

Breast cancer is one of the most common cancers globally. Unfortunately, many patients with breast cancer develop resistance to chemotherapy and tumor recurrence, which is primarily driven by breast cancer stem cells (BCSCs). BCSCs behave like stem cells and can self-renew and differentiate into mature tumor cells, enabling the cancer to regrow and metastasize. Key markers like CD44 and aldehyde dehydrogenase-1 (ALDH1), along with pathways like Wingless-related integration site (Wnt), Notch, and Hedgehog, are critical to regulating this stem-like behavior of BCSCs and, thus, are being investigated as targets for various new therapies. This review summarizes marker-dependent strategies for targeting BCSCs and expands on the challenges for the development of anti-BCSC drugs. We explore cutting-edge approaches like artificial intelligence (AI)-driven drug discovery and urge readers to seriously consider biological clocks and chronotherapy as experimental variables in drug discovery. Collectively, the task of cancer researchers is to overcome the many hurdles targeting BCSCs if we hope to tangibly improve breast cancer treatment outcomes and reduce mortality.

## 1. Introduction

Cancer is a leading cause of death worldwide, causing one out of every six deaths. In 2020, 2.26 million new cases of breast cancer were diagnosed, making breast cancer one of the most common cancers in the world [1]. The past 50 years have seen remarkable strides in screening for and treating breast cancer, with a 58% reduction in mortality [2]. However, breast cancer is far from cured, with 25–30% of these patients developing disease recurrence and dying from its spread through the body [3]. Mounting evidence supports that breast cancer stem cells (BCSCs) are a key player in recurrence and metastases for this disease; thus, the elimination of BCSCs is a top priority for drug discovery [4].

The breast tumor comprises a genetically heterogeneous mix of cells, with the majority being “bulk tumor cells”. A subset of these cells, BCSCs, contributes to aggressive progression, therapy resistance, and disease relapse due to their stem cell-like properties. More generally, cancer stem cells (CSCs), also called tumor-initiating cells, represent a subpopulation within tumors of various cancers that possess the unique ability to self-renew and differentiate into multiple cell types found in the cancer mass. The CSC concept has been well-established in the field of leukemias for a long time [5]. However, the CSC concept did not receive active attention in solid tumors until the 2003 breast cancer study by Al-Hajj et al. [6]. Although the CSC origin is still debated, the dominant thought on CSCs is that they drive tumors’ initiation, growth, and recurrence, much like how normal adult stem cells maintain tissue homeostasis. Similar to normal stem cells, CSCs are also regulated by transcription factors that promote pluripotency, such as Oct4, Sox2, Nanog, KLF4, and Myc, as well as intracellular signaling pathways, such as Wnt, Notch, and Hedgehog (Hh). Finally, CSCs are regulated by extracellular and microenvironmental factors, such as hypoxia [4]. All these components of CSC regulation are hot areas of active research.

### 1.1. Breast Cancer Subtypes and BCSC

Gene expression studies have classified breast cancer into five molecular subtypes: (1) luminal subtype A, (2) luminal subtype B, (3) HER2-overexpressing, (4) basal-like, and (5) normal breast-like [7,8]. Clinically, the type of breast cancer with the worst prognosis is the triple negative breast cancer (TNBC, Human epidermal growth factor receptor 2 (HER2)-negative/Progesterone receptor (PR)-negative/Estrogen receptor (ER)-negative) [9]. TNBC and basal-like subtype are different molecular classes of breast cancer with a high degree of overlap [10]. One intriguing aspect of tumor heterogeneity is that different breast cancer subtypes exhibit different proportions of BCSCs. TNBC and basal-like subtypes are enriched for BCSCs (CD44^+^/CD24^−^), while HER2^+^ tumors contain very few cells with this phenotype. Ricardo et al. [11] analyzed the expression of CD44/CD24 and ALDH1 in a panel of 466 invasive breast carcinomas, finding that basal-like tumors (76.5%) contained the highest percentage of cells with the CSC phenotype CD44^+^CD24^−/low^. Out of ALDH1-positive cases, 39.4% were also basal-like tumors (*p* < 0.0001) [11]. These observations support the notion that the enrichment of BCSCs in TNBC may be a culprit for the poor prognosis of TNBC. Yamamoto et al. further provided evidence of how NF-kB regulates the BCSC population in basal-like breast cancer [12]. As pointed out by Da Cruz Paula and Lopes [13], one must consider how the molecular diversity of breast cancer would define the nature of the BCSCs within a particular breast cancer subtype, as not all BCSC markers are expressed in all breast cancer subtypes. Different BCSC markers and different combinations of these markers may be restricted to a specific breast cancer subtype [13,14,15].

### 1.2. Cancer Stem Cells

**Figure 1:**
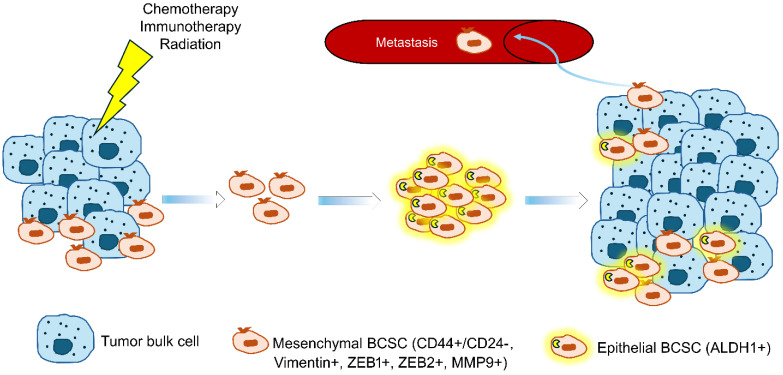
A schematic illustrating the concept of BCSC. A fraction of the original breast tumor contains quiescent mesenchymal BCSC cells (CD44+/CD24−, Vimentin+, ZEB1+, ZEB2+, and MMP9+) that behave like stem cells and resist many cancer therapeutics. In response to a stress signal such as cancer therapy, these CSCs can multiply and transform to the proliferative epithelial BCSC (ALDH1^+^) and restore the tumor bulk, a process called self-renewal and differentiation.

One hallmark of CSCs is their ability to self-renew and differentiate into multiple cell types, like normal stem cells. This dual capability enables CSCs to initiate and drive tumor development and to repopulate heterogeneous cancer cell populations critical for cancer progression [4,16]. CSCs can either undergo asymmetric division, where one daughter cell retains the self-renewing capacity of the CSC, and the other differentiates into a phenotypically distinct cancer cell with limited proliferative potential, integrating into the tumor bulk. Alternatively, CSCs can divide symmetrically, producing two daughter CSCs, which increases the CSC pool. Symmetrical division often occurs under stress conditions, such as cell loss during cancer treatments, facilitating excessive tumor growth and repopulation. Due to this ability to self-renew and differentiate, CSCs play a significant role in tumor progression and resistance to therapy. Evidence shows an increased CSC ratio after conventional treatment (Figure 1) [17].

In 2003, Al-Hajj et al. published a groundbreaking work that breast cancer cells bearing the phenotype of CD44^+^/CD24^−^ had enriched CSC properties, which set the foundation for studying CSCs in breast cancer and other solid tumors [18]. Markers such as these CD proteins are now the primary means of identifying, isolating, and targeting these cells. Specifically, BCSCs are commonly identified by immunohistochemical staining for CD44^+^/CD24^−^. Although they are not universal to BCSCs, these markers are used to classify BCSCs into two main subtypes: epithelial-like BCSCs and mesenchymal-like BCSCs. Epithelial-like BCSCs express high amounts of aldehyde dehydrogenase one enzyme family (ALDH1), another key BCSC marker, and are highly proliferative. They are often found in the center of the tumor and resemble a type of normal stem cell called luminal stem cells [19,20]. Mesenchymal-like BCSCs express CD44^+^/CD24^−^ and are stable, meaning they do not divide. They resemble a type of normal stem cell called basal stem cells [19] and are located at the invasive front of the tumor, which means they may play a role as the “seed” for metastasis. An accepted model is that the transition of BCSCs from the epithelial to mesenchymal state is needed to initiate metastasis. However, Fischer et al. argued against the concept that the transition of epithelial to mesenchymal state is essential for primary breast cancer metastasizing to the lung [21], while Ye et al. offered the counterargument [22].

To date, no U.S. FDA-approved therapy is designed solely to target or eliminate breast cancer stem cells across all subtypes of breast cancer. While some approved therapies, such as trastuzumab and lapatinib, may incidentally affect BCSCs, they were not developed with the primary intention of targeting these cells [23]. However, multiple experimental drugs are in clinical trials that are aiming to attack BCSCs by targeting an enzyme of a key pathway or surface marker, and this will be discussed in this review. Additionally, already-existing drugs have been investigated in research settings for their anti-BCSC effects. In 2009, the veterinary antibiotic Salinomycin was identified through high-throughput screening as a selective inhibitor of BCSCs, sparking widespread interest in targeting BCSCs [24]. However, to date, there is no clinical trial that is testing Salinomycin in breast cancer due to concerns with toxicity in humans [25]. Since then, numerous preclinical studies have focused on targeting signaling pathways (e.g., Wnt, Notch, and Hedgehog) and surface markers unique to CSCs. In particular, membrane proteins are an area of special interest since they are accessible on the cell surface and could serve as targets for monoclonal antibodies and other drugs [4]. Despite promising preclinical results, translating these findings into effective therapies for solid tumors has proven to be a formidable challenge. While there has been success in targeting CSCs of blood cancers [26], targeted therapy in solid tumors such as breast cancer is challenging because BCSCs make up such a small fraction of the tumor and are highly heterogeneous. During the process of conventional breast cancer treatment (e.g., chemotherapy, radiation, surgery), tumor cells with a high potential for proliferation are first identified and eliminated due to their high mitotic index. Still, CSCs continue to live because of their slow-dividing nature and expression of ABC efflux pumps (notably ABCG2), further promoting resistance [19]. This resistance can result in the initial shrinkage of the tumor followed by regrowth, often with a more aggressive phenotype.

Discovering an effective target of BCSCs could potentially overcome therapy resistance, prevent metastasis, and lead to more effective and lasting treatments for cancer patients. The following sections will delve into the current therapeutic strategies targeting BCSCs, as well as the challenges faced in this field, and recent advances in preclinical and clinical research. Among the many possible markers to target, our focus will be on protein markers, which exclude non-coding RNAs, biochemical markers in the mitochondria, and the tumor microenvironment (TME), which is defined as the local environment where CSCs live and are bathed in favorable factors that maintain CSC functions, such as specific cell types (e.g., stem cells and fibroblasts), cytokines, and growth factors [27].

## 2. Common Protein Markers of Breast Cancer Stem Cells

Overexpressed surface proteins on BCSCs compared to normal cells in the body function as a unique biomarker that researchers can exploit to target. Strategies that employ these surface markers can either inhibit the function of these proteins or serve as a “homing” marker to help improve the delivery of a cytotoxic agent. Targeting surface membrane biomarkers, such as CD proteins, makes a great deal of sense because of their accessibility. In recent years, work has been done to use novel immunotherapeutic approaches to target surface proteins, such as antibodies and chimeric antigen receptor (CAR) T-cell therapies. It is important to note that surface proteins are not the only target of recent novel discoveries; for instance, glycosphingolipids like GD2 have been recognized as a potential diagnostic marker of cancers of neuroectodermal origin (including breast). CAR-T-cell therapies that are specific to this marker have shown success in neuroblastoma and are under exploration for BCSCs [26]. Additionally, this list (Table 1) is not an exhaustive list of protein markers of BCSCs; we have chosen to specifically cover the markers that have the most active research in drug discovery and have successfully moved forward from preclinical research into clinical trials.

### 2.1. CD44^+^/CD24^−^ Are Classic Markers of BCSCs, but What Are They Exactly?

CD44 is a transmembrane glycoprotein that binds to a unique ligand: hyaluronic acid [33]. It plays a role in several biological processes but is notable for being a key mediator in the trans-endothelial migration of breast cancer cells during metastasis. Thus, CD44 is used to identify BCSCs and has also been explored as a potential target. CD24, also called heat-stable antigen (HSA), is a glycoprotein that plays several roles in the cell, including being a co-stimulatory molecule that helps activate CD4^+^ T cells. It is plausible that its expression would be nearly absent in BCSCs since lacking a CD4^+^ T-cell co-stimulatory molecule enhances immune evasion. Evidence from in vitro studies [34] demonstrated that the overexpression of CD24 was associated with the inhibition of stem-like properties in breast cancer cells, and another study [35] suggested that the lack of CD24 in breast cancer cells was associated with resistance against chemotherapeutics. Generally, CD24 is combined with CD44 as a classic biomarker of BCSCs [4].

### 2.2. CD133, Also Known as Prominin-1, Is a Transmembrane Glycoprotein Commonly Used as a Stem Cell Marker Due to Its Role in Suppressing Differentiation

Beyond this key role, CD133 also appears to be a master regulator of cell signaling and exerts a broad effect on many cell processes [36]. This protein is a hot area of pursuit for discovering anti-CD133 drugs because its overexpression in tumors is linked to poor prognosis, drug resistance, metastasis to lymph nodes, and other poor clinical outcomes for patients [4,29]. Therefore, CD133 is a common target in developing drugs against BCSCs.

### 2.3. EpCAM Is a Transmembrane Glycoprotein Expressed in Most Epithelial Cells, and It Mediates Cell–Cell Adhesion Independent of Ca^2+^, Which Is Unique and Different from Typical Epithelial Cadherins

Given its role in cell adhesion, EpCAM is involved in several biological processes such as migration, proliferation, and cell signaling, specifically through activating the Wnt signaling pathway. It is a notable and unique BCSC marker that deserves mention because it is expressed in circulating tumor cells. Circulating tumor cells are easier to detect than cells within the tumor mass because they can be identified from simple blood samples for markers like EpCAM. Although EpCAM is a CSC marker, we currently do not clearly understand the relationship between CSCs and circulating tumor cells [4]. Some have postulated that circulating tumor cells are CSCs that detached from the tumor and now circulate in the bloodstream, acting as “seeds” for metastases. This topic is discussed more in Section 3.

### 2.4. CXCR1, or C–X–C Motif Chemokine Receptor 1, Is One of the Receptors for Interleukin-8

This key communication molecule attracts neutrophils and is a key part of the body’s inflammatory response to infection or abnormal cell behavior (e.g., cancers). The IL-8–CXCR1 axis is worth mentioning because it is believed to be crucial for maintaining the stem cell-like features of breast cancer [37]; treatment with recombinant IL-8 led to enriched BCSCs, characterized by increased ALDH1 expression, mammosphere-forming efficacy, and greater invasive capacity [4].

### 2.5. DCLK1

Doublecortin-like kinase protein 1 (DCLK1) is a microtubule-associated protein with the C-terminal serine/threonine kinase domain. Now, DCLK1 is more appreciated as a CSC marker and is overexpressed in many types of cancer, including breast cancer [38,39]. This CSC marker was initially recognized as a microtubule-associated protein involved in neurogenesis and neuronal migration [40]. It was found to be overexpressed in intestinal tuft cells and stem cells, though there is a basal level of expression throughout the body [41]. Research supports the notion that the overexpression of DCLK1 in tumor cells will likely contribute to stemness and self-renewal [31].

Mechanistically, DCLK1-expressing tumors significantly enrich IL-6/STAT3 signaling, which exacerbates stemness traits. DCLK1 also plays a role in immunotherapy resistance by supporting immune escape mechanisms. It inhibits intra-tumoral cytotoxic T-cell infiltration, particularly in TNBC, further shielding BCSCs from immune surveillance. In a recent study, researchers successfully abolished the DCLK1-promoted malignant phenotypes of TNBC cells by inhibiting the IL-6/STAT3 pathway using an IL-6R antagonist, Tocilizumab, or a STAT3 inhibitor [42].

However, we should note that the literature on DCLK-1′s effect on cancers produced mixed results. Whether overexpression is associated with malignancy or poor prognosis in breast cancer appears to depend on the type and subtype of breast cancer. It has been demonstrated that the expression of DCLK1 was significantly increased in basal-like breast cancer tissues compared with normal mammary tissues, and DCLK1 overexpression could predict poor prognosis [43]. However, in another study, DCLK1 was positively related to favorable clinic–pathologic features for invasive breast cancers with neuroendocrine differentiation [44]. These contrasting reports illustrate that the precise role of DCLK1 in breast cancer has not yet been fully elucidated. However, more recent research appears to add evidence for DCLK1 playing an oncogenic role. In 2019, researchers found that DCLK1 promoted cell migratory and invasive abilities, typical of cancer metastasis. Their findings confirmed a metastatic-promoting role of DCLK1 in breast cancer, which was consistent with the role of DCLK1 in many other cancers [40]. Growing evidence supports that DCLK1 is highly enmeshed with cancer cell physiology. In basal-like breast cancer cells, a tumor-suppressive microRNA targeting DCLK1 was found to be decreased [43].

In addition to the growing research showing that DCLK1 overexpression promotes metastasis, evidence supports that DCLK1 is a worthy target for discovering drugs against CSCs [45]. One of the most striking features of DCLK1 is its tumor-specific expression pattern. Although this protein is universally expressed throughout the human body, its extracellular domain is usually absent or minimally expressed in normal tissues. Still, it is highly expressed on tumor cells, particularly CSCs. This selective surface expression makes DCLK1 highly accessible to targeted therapies while minimizing potential harm to healthy cells.

### 2.6. ALDH1

Aldehyde dehydrogenase-1 (ALDH1) is an endogenous enzyme that is found in the cytosol of normal cells that catalyzes the oxidation of intracellular aldehydes and actively drives stem-cell-like behaviors, and ALDH2 serves as a detoxifying enzyme with context-dependent roles in tumors [46]. Cytoplasmic variants of ALDH1 are also involved in retinoic acid biosynthesis, which plays critical downstream roles in cell differentiation and cell proliferation. This family of enzymes comprises 19 isoenzymes that play various important physiologic and toxicological functions, and among them, ALDH1A1 is a top-ranked hit that is recognized by the ALDH1 substrate metabolism assay (also called the ALDEFLUOR assay). It is an important biomarker in various cancers, including breast cancer [47]. Nearly all of the research on targeting BCSCs using ALDH1 only uses this enzyme as a marker for isolating and identifying BCSCs to measure the effect of the tested drugs. As mentioned above, the antibody-free fluorescent ALDEFLUOR assay is a popular method to characterize CSCs in various cancers.

In the normal metabolism of ethanol in the liver, ALDH2 converts the toxic intermediate metabolite, acetaldehyde, into the harmless compound, acetate. ALDH1 also has a detoxifying role that manifests as a remarkable resistance of BCSCs to chemotherapy and radiation; thus, enrichment of ALDH1 is a marker of drug resistance in breast cancer [48]. Specifically, enrichment of this enzyme is associated with the epithelial, highly proliferative subtype of BCSCs, whereas the mesenchymal-like BCSCs, which are not known to possess a high amount of ALDH1, divide more slowly and serve more as a “seed” to initiate metastasis. Aside from toxin metabolism, ALDH1 plays other roles in cells, which are discussed elsewhere [49]. For example, a 2021 study uncovered another potential mechanism by which ALDH1 contributes to BCSC function: ALDH1A1 lowers the intracellular pH in BCSCs, which promotes the phosphorylation of the intermediate protein TAK1. This, in turn, activates the transcription factor NF-κB, leading to increased secretion of GM-CSF. Elevated GM-CSF levels enhance the presence of immunosuppressive cells while reducing cytotoxic T cells, thereby facilitating immune evasion and promoting tumor progression [47]. In breast cancer, this enzyme is correlated with high histological-grade tumors, HER2 overexpression, and the absence of the expression of estrogen and progesterone receptors [16]. Clinically, ALDH1 is relevant because it can be used, along with other markers, to signal poor prognosis in breast cancer patients [50]. There are currently no clinical trials testing agents that directly inhibit ALDH1, but some studies use ALDH1 as a biomarker to identify and track BCSCs. ALDH1 activity is most assessed using the ALDEFLUOR assay, which measures enzymatic function in live cells. Alternatively, researchers may detect ALDH1^+^ cells using immunohistochemistry (IHC). For example, a Phase II trial evaluated the effects of neo-adjuvant Bevacizumab, a monoclonal antibody that inhibits VEGF and blocks angiogenesis, in combination with chemotherapy versus chemotherapy alone in breast cancer patients. In this study, the ALDH1 expression detected via IHC thus served as a marker to quantify the BCSC population. The study concluded that the increase in ALDH1^+^ tumor cells after bevacizumab-based chemotherapy was less than 5%, but these results were also observed with chemotherapy alone. Consequently, the trial was inconclusive in terms of the impact of bevacizumab on breast CSC cells [51]. A 2024 clinical trial investigating the neo-adjuvant humanized antibody Zilovertamab for targeting a constitutively active receptor tyrosine kinase in breast cancer cells was initially set to measure ALDH1 and CD133, BCSC surface markers, according to ClinicalTrials.gov. However, the final published manuscript made no mention of these markers being measured [52]. Despite these discouraging examples, a clinical trial testing a Notch signaling pathway inhibitor in BCSCs successfully reduced ALDH^+^ cells, along with other cancer stem cell markers such as CD44/CD24 and mammosphere-forming efficiency [53].

### 2.7. ABC Efflux Factors

ATP-binding cassette (ABC) transporters are membrane transporters that can pump various structurally unrelated and diverse small molecules out of the cells [54]. This superfamily is composed of numerous pumps, including P-glycoprotein (also called ABCB1; its expression is detected in many cancers), multidrug resistance proteins (including ABCC1, or multidrug resistance-associated protein 1), and breast cancer resistance protein (BCRP/ABCG2). Specifically, ABCA1 (a major transporter of lipids out of cells), ABCB1, and BCRP are key players in breast cancer chemoresistance, and ABCB1 and ABCC1 are important indicators of breast cancer prognosis [55]. Although they are expressed in both normal cells and cancer stem cells, it is believed CSCs express a high proportion of ABC transporters, allowing them to pump out chemotherapeutic agents, thereby serving as a drug resistance mechanism. One method of isolating CSCs is using a flow cytometry technique that is based on the efflux of a fluorescent dye called Hoescht 33342. This technique is aptly named the Hoescht side population (SP) method and has revealed that CSCs of all different tissues are highly enriched in ABC transporters [32]. Theoretically, the discovery of an agent that can inhibit the ABC efflux proteins of BCSCs could sensitize them to traditional chemo and radiotherapies. Indeed, various molecules have been identified that decrease ABC expression, reversing drug resistance by eliminating CSCs. However, adopting this strategy is risky and less popular than other strategies because ABC transporters exist in healthy tissues, so a systemic inhibitor could cause chemotherapeutic drugs to accumulate even in healthy tissues.

## 3. Current Strategies for Targeting Common BCSC Marker Molecules

### 3.1. Small Molecule Inhibitors

Research on small-molecule inhibitors of BCSCs typically focuses on suppressing signaling pathways rather than targeting surface protein markers. The protein markers on the cell surface are commonly used to help identify and isolate BCSCs instead of being targets for drugs. One reason why certain surface markers are a useful tool to quantify and characterize BCSCs is their accessibility. Moreover, the markers may be functionally involved in stemness behavior. That is, proteins like CD44 are active participants in key signaling pathways that are often in disarray in CSCs. From our review of preclinical studies on small-molecule inhibitors of BCSC surface proteins, we found instances where investigational drugs reduce the expression of markers like CD44 without a clearly defined mechanism. For instance, a recent study found that the plant compound Hinokitiol inhibits BCSCs in vitro by decreasing CD44 and transcription factor markers Nanog, SOX2, and Oct4 [56]. In BCSCs, Nanog partners with Oct4 to regulate the expression of SOX2, and it is understood that the upregulation of transcription factors depends on upregulated CD44 [57]. The authors posited that this small molecule generally inhibited BCSCs through the CD44/Nanog/SOX2/Oct4 pathway, but they did not detail how and where precisely the inhibition occurred. This example illustrates how the exact relationship between certain investigational drugs and CD markers is sometimes unclear due to the complex and poorly characterized interactions between the signaling pathways that drive stem-cell-like properties in CSCs.

In our present age, more resources are being devoted to bringing biologic immunomodulators into clinical trials. However, one primary surface protein inhibitor that was found clinically successful is Reparixin. Reparixin is an investigational small-molecule allosteric inhibitor of CXCR1, which has been shown to reduce BCSCs in human breast cancer xenografts in mice, both alone and in concert with chemotherapy [58]. Mechanistically, this molecule blocks IL-8 from binding to the CXCR1 receptor, which prevents a signal transduction pathway that involves the phosphorylation of proteins FAK, Akt, and eventually, the Wnt pathway (which promotes cell proliferation). In 2020, Goldstein’s group published the results of their Phase II clinical trial that examined the effect of Reparixin oral tablets on BCSC content in patients with HER-2-negative operable breast cancer [58]. Astoundingly, the group found that this drug safely decreases CXCR1^+^ cells. Reparixin also demonstrated a greater than 20% reduction of CD44^+^/CD24^−^ and ALDH1^+^ cells using flow cytometry; however, the authors could not confirm these results by using immunofluorescence due to the very low numbers of CSCs. Because ALDH1^+^ CSCs usually reside in the center of the tumor, while CD44^+^/CD24^−^ cells are found at the edge, and these cell phenotypes can transition between the types, sampling bias may have affected the results before and after treatment. Immunofluorescence showed very low numbers of CSCs, which made it challenging to quantify BSCSs visually. The RT-QPCR results also did not align with their flow cytometry data. Thus, the clinical relevance of a more than 20% reduction in CSCs is still unknown.

In another clinical trial that this group performed, which compared Reparixin plus Paclitaxel versus a Placebo plus Paclitaxel in women with metastatic TNBC, the authors ran into a similar issue [59]. At study entry, only 54 of the 123 patients provided an evaluable biopsy of CSCs of metastatic tissue, and positive biopsies were unevenly distributed in the treatment groups, limiting the power of the study to determine the predictive value of these markers. However, they used a novel method of evaluating anti-CSC: utilizing the time to new metastasis and the proportion of subjects progressing with new metastatic lesions. This method is plausible since the size of pre-existing tumors is likely reflective of chemotherapy on bulk tumor cells. At the same time, detecting new metastases may reflect the treatment effects on CSCs. However, this study had no statistically significant difference in time to new metastasis. Despite these negative results, Reparixin is still on the table for future clinical research because, in this study, there was an unbalanced amount of CSCs in the two groups, which is a confounding variable affecting the prognosis. Additionally, because CSC targets are so intertwined (i.e., CXCR1 activation leads to the upregulation of the Wnt signaling pathway), it is likely that targeting a single survival pathway is insufficient to make a clinical difference in patient outcomes.

### 3.2. Drug Delivery

Exploiting surface proteins as a strategy for optimizing drug delivery has shown more success for markers such as CD44 than strategies to create direct inhibitors of surface proteins. Notably, hyaluronic acid is the primary ligand for CD44 receptors. It has been used extensively to develop drug delivery systems that deliver approved cancer drugs, like paclitaxel and gemcitabine, to BCSCs, all of which have shown promise in sensitizing BCSCs to drugs [60,61]. There are various drug delivery systems, such as nanoparticles coated with hyaluronic acid, which can enhance endocytosis, nanogel–drug conjugates, or lipid-based particles like liposomes and micelles. This area of research, termed ‘nanomedicine’, and its application in drug delivery to CD44, is discussed at length elsewhere [62].

### 3.3. Immunotherapy

Moreover, advances in immunotherapy have opened a flood of research on targeting the surface proteins of BCSCs, such as chimeric antigen receptor (CAR) T-cell therapy, monoclonal antibodies, antibody–drug conjugates, and bi-specific antibodies. Nowadays, these cutting-edge approaches are most often used to aid drug discovery targeting surface proteins.

Monoclonal antibodies have been explored for various surface markers, such as anti-CD44, anti-Cadherin-3, anti-ErbB2/HER2, anti-CXCR1, anti-GD2, and anti-EpCAM [16]. Some agents show promise clinically, but overall, antibodies have been challenged on their own due to the heterogeneous nature of BCSCs. Since BCSC phenotypes vary and can also interchange between phenotypes, a single antibody by itself is unlikely to work in human patients. In addition, adverse effects have also gotten in the way of success. In a Phase I clinical study testing the safety of bivatuzumab mertansine [63,64]—an antibody targeting the CD44v6 isoform and conjugated to a cytotoxic agent—patients with metastatic breast cancer commonly experienced skin-related side effects due to CD44v6 expression in their skin tissue. However, the trial, along with others investigating the agent for different cancers, was discontinued after a fatal case of toxic epidermal necrolysis was reported in a parallel study.

**Figure 2:**
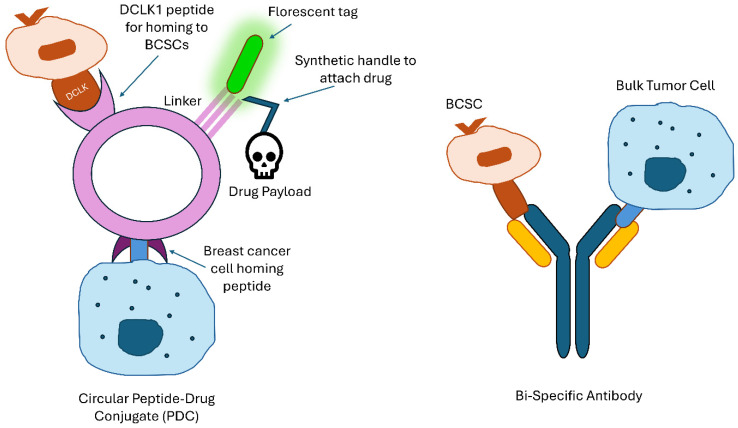
Design of bi-specific peptide–drug conjugate. On the left is our circular dual bi-specific PDC schematic designed to target both DCLK1 and breast cancer tissue. The PDC is constructed using two homing peptides: one is specific to breast cancer tissue (high-affinity) and one is for DCLK1 (lower-affinity), creating an “Uneven Bar” strategy. This structural feature enables the conjugated drug at the end of the synthetic handle to be more specific, with fewer off-target side effects, like the mechanism of bi-specific antibody drug conjugate (ADC) drugs. This dual homing approach allows preferential accumulation of PDCs in breast cancer tissue, maximizing delivery to breast cancer stem cells (BCSCs) while minimizing uptake by normal stem cells (NSCs) or other healthy tissue. This strategy leverages the differential expressions of DCLK1 and CD44 to improve therapeutic precision.

Another approach that could prevent off-target effects, such as these skin lesions, is bi-specific antibodies [65,66]. Catumaxomab was approved in Europe in 2009 for the treatment of malignant ascites, or fluid-containing cancer cells in the peritoneum of patients with EpCAM-positive carcinomas [67]. This antibody is bi-specific, meaning one antigen-binding site binds T cells, and the other binds to the EpCAM marker on tumor cells. See Figure 2 for an example of a bi-specific antibody. This antibody can increase desired effects and reduce off-target effects by bringing T cells and tumor cells together. This approach is promising, and recent research has tested the agent on breast cancer in vitro. However, no clinical trials have been conducted, possibly because the drug was pulled from the market after the manufacturing company went bankrupt [65,66].

From our review, EpCAM stands out as the surface marker that holds the most promise for targeted breast cancer therapy. A monoclonal antibody with moderate affinity for EpCAM—Adecatumumab—demonstrated its safety in clinical trials that were testing its administration with Docetaxel for patients with heavily pre-treated advanced-stage metastatic breast cancer [68]. As this antibody had only moderate affinity for EpCAM, it is possible that the toxic effects that would have been seen in high-affinity antibodies were avoided. However, due to the small sample size and the fact that stable disease (achieved in 33% of the patients) did not last for at least 6 months, its clinical benefits still cannot be determined, likely due to the heterogeneous nature of BCSCs. More research is needed to discover antibody-based drugs against BCSCs.

Chimeric antigen receptor (CAR) T cells, or genetically modified T cells, are a contemporary therapeutic strategy that has seen success for several cancers, especially blood cancers [69,70]. This novel therapy involves removing and enriching the patient’s T cells and then engineering them to express synthetic receptors that can recognize antigens unique to cancer cells, such as surface markers of BCSCs. After re-entering the body, CAR-T cells meet tumor cells, recognize their surface antigens, and activate an immune response to destroy these cells. One major challenge of CAR-T cells is localizing them to the tumor masses, which is why this therapy has seen significant success only in blood cancers, given that they do not exist as solid tumors. However, as mentioned above, EpCAM is a CSC marker that has also been demonstrated in circulating tumor cells (CTCs), and it is a marker that is correlated with tumor recurrence and metastasis. As a result, there has been much enthusiasm and fervor in researching EpCAM-directed CAR-T-cell therapies for various cancers, especially given the success in mice. One recent clinical study evaluated the safety of EpCAM CAR T cells in patients with EpCAM-expressing tumors of the epithelium (including gastric, colon, and rectal, but not breast [71]). The study’s main goal was to determine the safety of these transfusions. Although the authors deemed the treatment safe, making any conclusions on its efficacy was challenging, partly because the clinical response was more strongly correlated with the tumor burden at the start of the study than with the actual treatment of EpCAM CAR-T cells.

In conclusion, the review of clinical trials found that many did not provide promising data on BCSC reduction; only a small fraction seemed to impact BCSCs quantitatively. Most clinical trials have failed to translate preclinical success, particularly in targeting solid tumors like breast cancer. Ongoing innovations like bi-specific antibodies and personalized immunotherapies may overcome the current barriers.

### 3.4. DCLK1

Our current issue with standard chemotherapeutic agents is that they only kill a small proportion of CSCs and can end up harming normal tissue. CSCs and normal stem cells (NSCs) often share the same cell-surface markers or dysregulated signaling pathways that create stem-like behavior, so targeting these markers or pathways can result in putative off-target side effects. However, a novel approach to countering this problem is developing a unique drug delivery system that is bi-specific, or double-targeted, and takes advantage of the selective expression of specific markers like DCLK1. DCLK1 is ubiquitously expressed in the human body, so it is generally not a sensitive or specific marker for the sole use of targeting BCSCs. However, it can be utilized via a homing peptide strategy to maximize delivery that is specific to BCSCs. In 2016, a Chinese group did just that by developing a novel delivery system to CD44^+^ BCSCs by conjugating hyaluronic acid (HA) and the DCLK1 monoclonal antibody to the surface of nanoparticles [71]. HA is a recognized ligand for CD44, in which the binding of this ligand facilitates endocytosis. However, using CD44 alone as a marker to target has some limitations, such as being widely expressed in normal epithelial cells, having a short recycling half-life, and being easily saturated. As a result, the exploration into dual-targeted drug delivery systems that incorporate more tumor-specific markers, such as DCLK1, to enhance selectivity and therapeutic precision, is a promising strategy for the future of drug development against BCSCs. By adding DCLK1 to this drug delivery system, the research team discovered that the nanoparticle allowed them to discern between CSCs and NSCs. Notably, our lab has conducted research using unique circular homing peptides with selective, strong affinities that bind to different receptors on the surfaces of breast cancer tissue (Figure 2) [72]. Our bi-specific peptide–drug conjugate (PDC) utilizes two homing mechanisms to minimize off-target binding, such as to neural or intestinal tissue. This “Uneven Bar” strategy is expected to lead to an accumulation of PDC in breast cancer tissue, enabling the DCLK1 homing peptide to bind to BCSCs. Thus far, we have successfully created a drug delivery system that targets BCSCs specifically, putatively avoiding off-target toxicity of the PDC. One unique attribute of the PDC that sets it apart from existing homing peptides is its circular breast tissue homing peptide design, which enhances its pharmacokinetic and pharmacodynamic profiles. Additionally, the addition of a fluorescent tag will enable visualization of the drug using confocal fluorescence microscopy [73]. A DCLK1 homing peptide was proposed to act as a selective affinity binding navigator and function as a blocker/neutralizer to interrupt the pro-cancer activities of DCLK1. Our unique PDC is designed as a dual bi-specific towards DCLK1 and breast cancer tissue. This structural feature enables the conjugated drugs to be more specific, with potentially fewer off-target side effects.

### 3.5. ALDH1

Although work aiming to target ALDH1 in the BCSC field is not common, there exists one FDA-approved agent that is already used in patient care to irreversibly inhibit ALDH1A1. Disulfiram is a second-line treatment for alcoholism, which produces an irreversible inhibition of ALDH, thus leading to the accumulation of the toxic acetaldehyde metabolite of ethanol. This toxic accumulation creates undesirable effects that lead to an aversion to drinking alcohol. Repurposing this drug has been considered to inhibit CSCs. Recent data show that Disulfiram potently inhibits CSCs of various cancers, surprisingly through various mechanisms, such as inhibiting stemness-associated transcription factors (Sox, Nanog, and Oct) and modulating ROS generation. For example, in vitro, Disulfiram complexes with copper ions, which downregulate the NF-κB-stemness gene pathway, and these complexes also induce reactive oxygen species (ROS) and endoplasmic reticulum (ER) stress, which increases cell death of BCSCs. In vivo, the combined treatment of radiotherapy and Disulfiram significantly inhibited mammary primary tumor growth and lung metastasis in mice [74]. Due to the surprisingly varied roles that ALDH1 plays in BCSCs, recent discussions have advocated for more work to authenticate ALDH1 as a genuine therapeutic target in solid tumors [75]. Outside of Disulfiram, there is little research on BCSCs that aims to directly target ALDH; however, there has been some work that indirectly inhibits the enzyme. In 2023, it was found that the cancer-associated protein KK-LC-1 promotes the stemness of BCSCs by binding to and inducing the degradation of FAT1, an upstream regulator of the Hippo signaling pathway [76]. Degradation of FAT1 compromises the Hippo pathway, leading to an increased transcription of stemness-related genes, including ALDH1A1. This results in a higher proportion of ALDH^+^ cells, which are associated with more aggressive tumor features. Using a virtual screening approach, the research team identified a small-molecule inhibitor (Z8) that disrupts the KK-LC-1/FAT1 interaction. This small molecule (Z8) preserves FAT1 stability. It also maintains Hippo pathway activity, which illustrates the capacity of Z8 to reduce ALDH1A1 activity.

To conclude this section, we discuss another hot area of research that explores novel techniques for identifying and isolating BCSCs using ALDH1 and methodologies beyond the standard ALDEFLUOR assay. Two that have been developed in recent years are long-acting fluorescent probes and reporter constructs.

In 2022, a Japanese group developed a small-molecule probe named C5S-A, which becomes highly emissive upon being turned over by ALDH1A1, creating a stronger signal than the ALDEFLUOR assay [77]. Specifically, the signal-to-noise ratio for C5S-A was 8.3, which is substantially higher than that of ALDEFLUOR, which is 1.9. One additional advantage of this new probe is that the fluorescence from a single staining remained observable for more than three days, which opens doors for investigating chronological changes during drug treatment.

In addition to chemical probes, reporter constructs, such as fluorescent and luciferase reporters, are emerging as a novel way of identifying CSCs for non-surface marker proteins [78,79]. The current primary mode of detection of CSCs is to use antibodies against surface proteins, which may be cumbersome, costly, and difficult to conduct when screening drugs. Additionally, they do not work against intracellular markers. The biggest advantage of reporter constructs is that they are a cheaper, more efficient way of large-scale screening of inhibitors that also address the heterogeneity of BCSCs (which is to be addressed in depth in Section 2 of this review). In other words, CSCs exist in various stages: self-renewal, differentiation, dormancy, and proliferation. For example, CSCs marked with ALDH1A1 are more proliferative, but dormant CSCs show greater activity of the Oct4 promoter. Since BCSCs are so heterogeneous, a dual-reporter system that tracks both ALDH1 and Oct4 would be a more comprehensive way to capture the variety of CSC states than a single reporter. Pluripotency transcription factors and enzymes like ALDH1A1 are great candidates for reporter constructs because they impart stemness depending on their level of expression. Thus far, there are reports for preclinical studies using reporters for OCT4, SOX2, NANOG, ALDH1A1, and ABCG2 to screen for drugs targeting BCSCs [80].

### 3.6. ABC Efflux Factors

Some agents are already in use for various conditions that can also block the ABC transporter MDR1, but their clinical efficacy is overshadowed by their toxic effects. Examples of these so-called “first-generation modulators” include calcium channel blockers, antiarrhythmics, immunosuppressants, and anti-estrogen compounds. Subsequent second-generation and third-generation modulators have been studied, with each generation showing more selectivity [29]. The concept of natural products, such as flavonoids, terpenoids, and alkaloids (from plants, marine, and microorganisms), has been coined as “fourth-generation MDR modulators,” though this development is still ongoing [81]. The third-generation ABC modulators include elacridar, laniquidar, zosuquidar, and tariquidar. Tariquidar (XR-9576) has the most data supporting its efficacy as a selective ABCB1 inhibitor, where, in healthy human volunteers, the brain uptake of ABCB1 substrates showed approximately five- and three-fold increases with a concurrent administration of tariquidar, respectively [82].

Additionally, exciting research is occurring in small-molecule tyrosine kinase inhibitors that inhibit ABC transporters. Coincidentally, ABC transporters happen to have two binding domains that can serve as ATP-binding pockets, such as on tyrosine kinases. Agents include Lapatinib, Nilotinib, Linsitinib, and Mastinib. Of these, Lapatinib has shown to be of potential promise. As a dual tyrosine kinase inhibitor, this agent’s intended mechanism of action is to interrupt the HER2/neu and EGFR pathways, and was approved to be prescribed for breast cancer and other solid tumors by the FDA in 2007. A study showed that lapatinib dose-dependently increased the efficacy of various cancer drugs, docetaxel, paclitaxel, vinblastine, and vinorelbine in HEK-MRP7-2 cells, enhancing these drugs’ accumulation significantly by blocking their efflux. Clinical trials have also shown that Lapatinib can improve the efficacy of chemotherapeutics in breast cancer patients [83].

However, those clinical trials were from 2008, and no notable research has been done on this topic recently, likely due to the impracticality of the clinical application of ABC transporter inhibitors. As mentioned before, these agents can potentially induce serious off-target effects. Additionally, although the increased expression of ABC transporters could render CSCs resistant to drugs, numerous other factors can either inhibit or increase the expression of ABC transporters. Most notably, microRNAs play a key role in regulating the expression of ABC transporters and, thus, the chemoresistance of BCSCs. Drug development trends in the future for compounds targeting ABC transporters may consider using or targeting microRNAs rather than the enzyme itself. More discussion on the limitations of targeting ABC transporters alone in breast cancer can be found elsewhere [82].

## 4. BCSC Targets in Signaling Pathways

**Figure 3:**
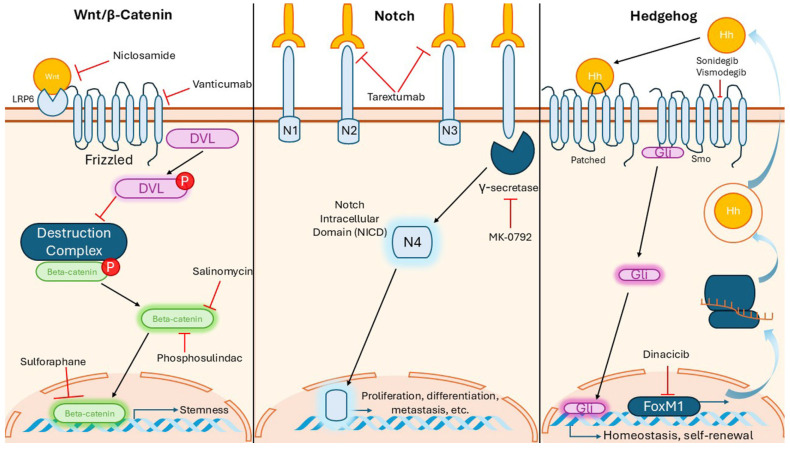
Simplified diagram depicting the basic signaling pathways of Wnt/β-catenin, Notch, and Hedgehog. Experimental small-molecule inhibitors and antibodies that were mentioned are also illustrated here.

Because normal stem cells and CSCs must renew themselves, it is reasonable to assume that they share some molecular mechanisms that regulate this critical stem cell function. These signaling pathways enable BCSCs to have a greater capacity to proliferate and tolerate hostile environments [84]. However, in both CSCs and cancer cells, these pathways are usually dysregulated in some way that promotes pathogenic proliferation and self-renewal. As a result, it is popular to target certain receptors and enzymes in the signaling pathways that are critical for the self-renewal function of CSCs. An agent that shows promise in targeting BCSCs is defined as reducing BCSC markers and CSC characteristics, such as sensitizing cells to radiation. Most of the agents that have successfully made it into clinical trials are usually signaling pathway inhibitors. Here, we briefly describe (i) the most targeted pathways, such as Wnt/β-catenin, Notch, and Hedgehog (Figure 3), and (ii) a selection of promising agents that target BCSCs. Please note that our discussion of signaling pathways is far from exhaustive. There are other dysregulated pathways that contribute to BCSC characteristics. These pathways include the TGFβ/Smad pathway, protein kinase Cα, NF-kB pathway, P13k/Akt/mTOR, and KFL4, which are discussed elsewhere [29].

Mutations and the dysregulation of the Wnt/β-catenin pathway are infamously linked to numerous cancers, but they are also implicated in promoting self-renewal characteristics in BCSCs. Its mechanism involves secreted Wnt ligands binding to Frizzled receptors and activating a cascade that is important for stem cell development, as well as maintenance and regeneration in normal cells. For BCSCs, it has been shown that increased activation of this signaling pathway significantly contributes to BCSCs’ self-renewal and therapy resistance. Various studies show that inhibiting this pathway suppresses BCSC proliferation [4,84]. However, successful clinical translation is not very robust. For example, Vantictumab is a monoclonal antibody that binds to the frizzled receptor to inhibit Wnt signaling (Figure 3). Phase 1 clinical trials wrapped up in 2020 showed promising efficacy, but unfortunately, its use was discontinued by the end of the study due to a high rate of fractures in the treated patients. Therefore, the study was closed due to the risks outweighing the potential benefits [85].

Another initially promising agent that has not lived up to the hype is the first compound to be identified as a selective inhibitor of BCSCs in 2009—the veterinary antibiotic, salinomycin. This agent was shown to reduce the proportion of CSCs by more than 100-fold relative to paclitaxel, and treatment of mice inhibited mammary tumor growth [24]. Salinomycin mechanistically targets a wide spectrum of targets within the cancer cell, with one of them being the downregulation of β-catenin, thus inhibiting Wnt signaling (Figure 3). However, to date, there is still no registered clinical trial for salinomycin [86]. Its use has been documented in case studies of patients with cancer showing efficacy; however, more work would need to be done to study any potential toxicities of salinomycin in humans. It is intriguing to note that combination therapy with salinomycin has been shown to achieve promising results for breast cancer in general [25].

Overall, agents that target the Wnt/β-catenin pathway in BCSCs show promising preclinical activities, but so far, there are no FDA-approved Wnt signaling inhibitors [87,88]. For example, the anti-helminth drug niclosamide successfully showed cytotoxicity to BCSCs by reducing levels of downstream receptors in the Wnt pathway, like LRP6 and β-catenin, both in vitro and in tumor xenografts (Figure 3) [89]. Additional clinical data proving the safety and effectiveness of niclosamide remain to be demonstrated in the treatment of colorectal cancer [90]. Recent studies since the discovery of niclosamide from drug screening in 2013 further support the anti-CSC promise of this anti-helminth drug, but we have not found any clinical trials that have tested this agent in breast cancer patients [91]. Other agents with preliminary data of inhibiting BCSCs include the nonsteroidal anti-inflammatory derivative phosphoulindac and the natural plant compound sulforaphane, which is derived from broccoli sprouts [29].

Notch is a tremendously popular target for BCSCs [92,93]. This transmembrane system in all animals regulates stem cell development and normal cell maintenance. Specifically, many anti-CSC agents target the enzyme γ-secretase. Furthermore, γ-secretase cleaves one of the transmembrane Notch ligands, leading to an intracellular cascade that ultimately activates a series of transcription factors. Notch is relevant for BCSCs because it is reportedly overactive in BC and promotes resistance against chemotherapy and radiotherapy in breast cancer and BCSCs. The data support the Notch4 ligand as one of the most practical and effective molecules one to suppress in preventing breast cancer recurrence originating from BCSCs [94]. Whether targeting Notch alone or in combination with inhibitors targeting other pathways, several promising inhibitors have emerged [95,96].

In 2009, Merck developed MK-0752, a γ-secretase inhibitor that reduced BCSCs in preclinical studies. A 2013 clinical trial treated 30 patients with advanced breast cancer using escalating doses of MK-0752 combined with docetaxel (Figure 3) [97]. The results showed that clinically meaningful doses of both drugs were achievable with manageable toxicity, along with preliminary evidence of efficacy. Serial tumor biopsies revealed a decrease in CD44^+^/CD24^−^ cells, ALDH1^+^ cells, and mammosphere-forming efficiency [53]. Other investigational agents and biologics have shown promise in targeting BCSCs but have faced challenges in attempts to move them forward clinically. For example, the monoclonal antibody Tarextumab, an inhibitor of Notch 2 and 3 ligands (Figure 3), demonstrated potential in Phase I studies for various cancers. However, it had to be discontinued after Phase II trials because they failed to improve patient outcomes [97].

Due to the challenges of translating preclinical findings into clinical success, a popular strategy is repurposing existing FDA-approved drugs. One such example is Metformin, an anti-diabetes drug that has received significant attention for its ability to target BCSCs through multiple signaling pathways, including Notch and PI3K/Akt/mTOR. In 2019, a Korean research team found that encapsulating Metformin with the anti-cancer drug Herceptin (Trastuzumab) into a liposome effectively targeted BCSCs in both in vitro and mouse models [98]. Despite these promising findings, a 2022 meta-analysis of randomized controlled trials evaluating Metformin in combination with chemotherapy and endocrine therapy for breast cancer patients found inconsistent results [99]. As a result, Metformin has yet to demonstrate robust clinical efficacy in breast cancer treatment, highlighting the need for further research.

The Hedgehog (Hh) pathway is a fundamental pathway that is highly critical for tissue homeostasis and self-renewal [100]. Its activation is implicated in several cancers and disorders [101]. For example, dysregulation of its secreted ligand Sonic Hedgehog (Shh) causes limb deformities, and mutations in its transmembrane receptor Patched (PTCH) are implicated in basal cell carcinoma. Notably, abnormal upregulation of the Hh is found in BCSCs. Among the cancer agents that serve to inhibit the Hh pathway, the most popular target is the enzyme smoothened (SMO), a transmembrane protein that works downstream of PTCH. Several well-known Hh inhibitors that have entered clinical trials for BCSCs include Vismodegib and Sonidegib (Figure 3), both of which are oral inhibitors of SMO and are already approved to treat basal cell carcinoma. These SMO inhibitors are in clinical trials for efficacy in various cancers. There are some recent trials examining their effect in breast cancer.

Vismodegib treatment on Tamoxifen-resistant xenografts blocked tumor growth in mice. It was approved for clinical trial use in combination with other chemotherapeutics in TNBC [102], but there has sadly been no update on the clinical trial since 2017 (ClinicalTrials.gov ID NCT02694224). Second in the class of SMO inhibitors after Vismodegib is Sonidegib. In a phase 1b clinical trial on ten patients with advanced TNBC, combining Sonidegib with Docetaxel was shown to be safe, and it exerted antitumor activities in three patients [103]. Although the sample size was too small to make any firm conclusions, this clinical trial does support continued research into using SMO inhibitors to block recurrence and metastasis in breast cancer.

Interestingly, another recently discussed Hedgehog inhibitor is Dinaciclib, a small-molecule inhibitor of cyclin-dependent kinases (CDKs) that is currently being investigated in clinical trials for various cancers, including breast cancer. While its primary mechanism involves CDK inhibition, a 2022 study found that Dinaciclib also suppresses the stemness characteristics of breast cancer cells in two cell lines [104]. Specifically, it reduced the expression levels of CD44, ALDH1A1, and the embryonic stem cell markers Oct4, Nanog, and Sox2. The study further revealed that Dinaciclib inhibits an upstream regulator of the Hedgehog signaling pathway—FoxM1—which is a key transcription factor (Figure 3). These findings support the continued clinical development of Dinaciclib as a potential treatment for breast cancer.

In conclusion, signaling pathways remain the most popular target for drug discovery strategies. Many agents are currently being investigated, and promising results have been shown in inhibiting BCSCs in the preclinical setting. However, in this review, we have specifically highlighted strategies that have successfully led to the development of agents that have advanced into clinical trials.

## 5. Challenges of the Existing Marker-Dependent Drug Discoveries Against Breast Cancer Stem Cells

Inherent resistance of CSCs to drugs is a major challenge in discovering drugs that target CSCs. CSCs of all cancers have increased expression of DNA repair proteins, ABC transporters, and ALDH1 enzyme, which reduce reactive oxygen species and, thus, apoptosis [4]. However, the biggest challenge of targeting BCSCs so far is addressing tumor heterogeneity, while avoiding off-target effects, such as accidentally targeting normal stem cells. The unique stemness-associated transcription factors and signaling factors expressed by BCSCs are shared by normal stem cells. Therefore, existing therapies have the issue of specificity for BCSCs [4,16]. This challenge stems from an over-reliance on specific BCSC markers for therapeutic targeting. While certain markers can provide a means to isolate and attack BCSCs, their expression can be influenced by various factors, including past treatment history and the tumor microenvironment (TME), which is the local microenvironment of the CSC, and can be influenced by multiple factors such as specific cell types (stem cells, fibroblasts), cytokines, and growth factors. The variability of BCSC phenotypes in tumors leads to concerns surrounding investigational therapies that are designed to target one or two markers. Because of the many permutations of markers, these experimental drugs may inadvertently spare other BCSCs or even damage normal stem cells with similar markers. Consequently, BCSC drug discoveries that can differentiate between BCSCs and normal cells without causing toxic off-target effects remain a formidable task.

Aside from differentiating BCSCs from normal stem cells, another main challenge that has been introduced is the heterogeneity of BCSC markers [105], which can vary not only between patients but also within different regions of the same tumor. For instance, some BCSCs may express specific surface markers like CD44 or ALDH1, while others may not. Part of the variability of BCSCs can be explained by the existence of different subtypes of BCSCs, which differ in location, behavior, and marker expression. It is important to be aware of these different subtypes because they can respond differently to therapeutic agents. For instance, targeting DCLK-1 in TNBC mesenchymal-like cells with a DCLK1 inhibitor and chemotherapeutic doxorubicin effectively kills mesenchymal-like cells, yet such a combined treatment did not show a significant effect in basal-like cells. In that study, the team hypothesized that the insignificant effect on basal-like cells was possibly due to their inherent insensitivity to chemotherapy and low DCLK1 expression [42]. This story illustrates how the heterogeneity of BCSC populations makes eradicating them tricky. For this very reason, detecting them is also challenging. Unfortunately, BCSCs occur in very low concentrations, and even if a tumor biopsy were to capture the whole spectrum of cancer cells successfully, the technique used to detect common markers like CD44, CD24, and ALDH1 by the immunohistochemistry (IHC) method is semi-quantitative and can be subject to variability between different people interpreting the results. For instance, a 2024 phase II study of Ruxolitinib, a JAK1/2 inhibitor, and Paclitaxel for inflammatory TNBC, aimed to inhibit CD44^+^/CD24^−^ BCSCs, as the JAK2/STAT3 pathway was required for their growth [106]. However, while analyzing the data, the researchers had difficulty assessing marker pSTAT3 levels of the stem cell-like cells by immunofluorescence due to profound intratumor heterogeneity, and it was difficult to identify which patients’ tumors had undergone a meaningful biologic response to Ruxolitinib. While this heterogeneity in pSTAT3 levels could be due to technical limitations, the researchers reasoned it was more likely due to the substantial heterogeneity found in inflammatory breast cancer tumors. As an alternative, flow cytometry can be used based on the expression of these markers to quantify and sort BCSCs. It may be combined with the aldehyde dehydrogenase activity assessment, an ALDEFLUOR assay. But this flow cytometry approach requires fresh and viable single-cell suspensions that are derived from tumor tissue. CSCs are typically present at a very low percentage of the tumor; their markers overlap with those of other CSCs and even NSCs, and the absence of some markers is used to classify certain CSCs. This heterogeneity not only affects the efficacy of targeted therapies but also contributes to the phenomenon of tumor recurrence, as a subpopulation of BCSCs may evade treatment and repopulate the tumor.

Finally, some studies were conducted in vitro, which does not accurately reflect the genuine tumor microenvironment of BCSCs. A living organism is a dynamic, complex system that in vitro cell lines just cannot fully emulate. In vitro studies often have short experimental durations, which may not capture the long-term effects of the drug or the development of drug resistance in CSCs over time. Patient-derived xenografts (PDX) [107] are an experimental system that potentially minimizes these liabilities. However, PDXs are expensive and have a time-consuming preparation protocol. Other innovations, like patient-derived organoids (PDOs), are promising in vitro models. They mimic the original cancer tissues’ TME, gene expression, and cell types [108]. Recently, researchers discovered that LFS-01, a sulforaphane-derived compound from traditional Chinese herbal medicine, is a highly potent inhibitor that selectively eliminates BCSCs. Its activity is derived from blocking a nuclear export protein that is responsible for retaining survivin, a key protein that prevents the activation of downstream stemness regulators [109]. The researchers applied this drug to TNBC PDOs and found it could significantly remove cancer cells compared to approved cancer drugs like Olaparib, and it enhanced the cytotoxic effects of Epirubicin and Docetaxel, which bolsters the preclinical evidence for this compound as a therapeutic agent to deplete BCSCs. To obtain more comprehensive evidence to guide clinical studies of BCSC-targeting drugs, it is essential to move towards the use of models that resemble the tumor microenvironment. There is currently a lot of variability in the results of BCSC-targeting therapies, which underscores the need to improve the translation of findings from preclinical studies to clinical.

In conclusion, targeting CSCs effectively requires addressing a slew of obstacles, such as the heterogenous assortment of cells, off-target effects on normal tissue and normal stem cells, and determining how to inhibit the signaling pathways, as they all interact with each other like an interconnected web. Even if agents show promise in vitro, BCSC research into clinical success remains challenging. Tumor heterogeneity plays a significant role, but logistical hurdles also contribute. For instance, in a phase II clinical trial that sought to evaluate the effectiveness of combining lapatinib ditosylate (a tyrosine kinase inhibitor) with radiation therapy, a secondary objective was to assess the impact of this combination on BCSCs by measuring relevant markers (ClinicalTrials.gov ID NCT01868503). But funding shortages ultimately limited its ability to investigate the BCSC markers as originally planned. This story illustrates how BCSC targeting may be an afterthought in the development of drugs that target tumor cells in general. Overall, the complexity of BCSCs has created significant barriers to translating promising in vitro findings into clinically effective therapies.

## 6. Future Directions in Targeting Breast Cancer Stem Cells

### 6.1. Circadian Therapy

A growing body of evidence supports that biological clocks are intertwined with cancer biology [110]. Both our daily circadian rhythms and the estrus cycle (menstrual cycle in humans) should be parameters that researchers consider during the drug discovery process, and there should be a more urgent attitude toward utilizing chronotherapy to maximize the therapeutic effects of breast cancer treatment. Circadian clocks are a biological timekeeping system that can anticipate the daily changes in light exposure and temperature and, as such, organize physiology and behavior around this 24 h rhythm. These rhythms are autonomous and can function independently due to the way they are set up: by interlocked transcriptional and translational feedback loops between genes and their protein products. Among the most prominent players in clock genes are the master genes Clock and Bmal1 [111]. These genes form a heterodimer and act in unison to activate the expression of their repressors: Period (Per 1–3) and Cryptochrome (Cry 1–2). After a defined time delay, Per and Cry proteins form protein complexes and translocate back into the nucleus to suppress the activity of their own activators (Clock and Bmal1), resulting in an oscillating rhythm that lasts 24 h per cycle. These major clock genes control every aspect of physiology and cell activity. Emerging evidence has implicated the circadian clock and clock genes in regulating the stemness and functional features of CSCs. Prior studies examining clock genes in various cancers suggest that these genes may play either oncogenic or tumor-suppressive functions, depending on the cancer types and contexts [110]. Aside from the central clock, primarily controlled by melatonin, our peripheral clocks that control processes like metabolism and immune function are strongly influenced by glucocorticoids, which reach peak concentration levels in the early morning and repress melatonin secretion around this time. It is plausible that circadian dysregulation can lead to cell cycle disorders and cancer, since the cell cycle is driven by factors like nutrient availability, a product of the glucocorticoid-coordinated metabolism process. Given this crosstalk that is occurring between cancer and the circadian clock, it is apparent that circadian biology is an emerging, but increasingly important, factor in cancer research.

In mice, forced chronic jet lag had significantly increased tumorigenicity and shortened survival compared to the controls. Humans bear a wide variety of confounding factors like race, local environment, and culture, which would make it difficult to replicate these results and prove definitively that circadian misalignment directly causes cancer. Regardless, the accumulating evidence gives enough reason to at least consider the role of chronic circadian misalignment, such as shift work, in contributing to an increased rate of cell mutations that could lead to cancer. There has been one landmark study examining chronotherapy and BCSCs in 2018 [112], which is when a Japanese group found that the ALDH3A1 enzyme exhibited cyclic oscillations of expression with peak abundance at the time point roughly 14 h after the lights turned on in a light/dark cycle (ZT14). Administration of a general ALDH inhibitor at ZT14, when the number of ALDH3A1^+^ cells was abundant, elicited dramatic antitumor and anti-metastatic effects on TNBC cells, suggesting that choosing the optimal drug dosing time is of potential importance in treating TNBC by targeting BCSCs. Aside from utilizing chronotherapy as a therapeutic approach, the group found that the CLOCK level was downregulated in ALDH^+^ cells in the murine 4T1 breast cancer cells, and overexpression of CLOCK markedly inhibited the tumorigenicity and invasive capacities of these breast cancer cells. These findings only illustrate how clock genes are involved in the tumorigenicity and invasiveness of breast cancer cells in vitro. However, invigorating news over the past year revealed that the targeting of CSCs’ clock genes is a cutting-edge strategy, both in vivo and potentially in humans. This past year, a clock-targeting drug aiming to treat glioblastoma (a brain cancer with a dismal prognosis) passed phase I trials, and the research team just finished enrollment for phase IIb trials [113]. Excitingly, this drug’s mechanism of action targets CSCs of glioblastoma directly rather than as an add-on to fighting bulk tumor cells. The master genes BMAL1 and CLOCK are both essential for the survival of glioblastoma CSCs, so the researchers developed a Cry activator, SHP1705, which helps inhibit BMAL1 and CLOCK transcriptional activity. They discovered that CSCs were extremely and selectively sensitive to their compound in vitro, and in vivo studies revealed that the drug delayed the glioblastoma tumor growth rate and increased survival in mice with patient-derived xenografts. Excitingly, this drug was not only safe and tolerated in healthy humans, but it also did not significantly alter the expression of the other clock genes, which the researchers hypothesized was due to the strong effects of external entrainment signals like light and temperature. Broadly speaking, SHIP1705 is the first clock-targeting compound to complete a phase 1 clinical trial, making this achievement particularly extraordinary.

Overall, the concept of biological rhythms has introduced a novel approach for treating cancers. Recently, we have learned that circulating tumor cells (CTCs) preferentially invade into blood vessels during sleep in both human and mouse breast cancer models [114]. This discovery helped characterize the temporal nature of breast cancer metastasis and was further evidence that showed it is not necessarily true that CTCs are constantly shed from growing tumors. In this 2022 study [114], Diamantopoulou and her colleagues confirmed these observations by using a single-cell RNA sequencing analysis of CTCs, which revealed a marked upregulation of mitotic genes exclusively during the rest phase in both patients and mouse models. They also found that key circadian rhythm hormones such as melatonin, testosterone, and glucocorticoids dictate these patterns. When the team altered the light/dark cycles to various ratios, they found a constant pattern of increase in CTC counts during the rest phase in mice, underscoring the key role of light exposure in CTC activity. Finally, they found that abolishing the master regulator gene of the circadian clock, Bmal1, also abolished CTC movement into blood vessels. This remarkable finding provides even more reason for time-controlled research and treatment of metastasis-prone cancers like breast cancer.

### 6.2. CTCs vs. CSCs

One important avenue for further research is distinguishing between CTCs and CSCs. The relationship between these two types of cells is not clearly elucidated, although some CTCs capable of seeding metastasis are enriched in CSC states in some experimental models [115]. From our review, there is likely more reason than not that CSCs are related to CTCs, as the two share similar markers. CSCs possess reduced adhesion, partially allowing them to break off the original tumor and metastasize. This process, called epithelial to mesenchymal transition (EMT), is characterized by a change in the ratio of adhesion molecules, specifically an upregulation of N-cadherin and downregulation of E-cadherin [116,117]. Additionally, prior research has noted that the BCSC marker CD44 is a standard marker of CTCs of various cancers [118]. This area of research is in dire need of new methods to identify CSCs in tumors because only a tiny fraction of tumoral cells are CSCs. For example, as aforementioned, in a clinical trial for the efficacy of Reparixin (a CXCR1 inhibitor) in HER-2-negative breast cancer, CSC markers ALDH and CD24−/CD44 decreased by more than 20% in several of the participants. However, the team could not confirm those results using immunofluorescence due to the very low number of CSCs [58]. Traditionally, it has been challenging to characterize CSCs using immunofluorescence alone because of the subjective nature of analysis and the small number of cells in the tumor. Therefore, it is imperative to elucidate whether CTCs are identical to CSCs. Once CSCs are in circulation, identifying and targeting them may be easier. However, they face similar challenges, such as a low number of CTCs that can be isolated from peripheral circulation [119].

The abovementioned research discussing CTC intravasation at night could provide helpful future insight into the temporal nature of CSCs in metastasis. But there is still a lot to be elucidated. Currently, the applications of circadian medicine and CTCs are unknown. Studies using in vivo flow cytometry (a technique to detect and quantify cells in circulation without drawing blood samples) have revealed mixed results. Most research shows a constant CTC number in mice over the days and hours, or small fluctuations that do not match a daily cycle [120,121]. However, other published studies revealed circadian oscillations [122,123]. Our understanding is that, unfortunately, the circadian nature of CTCs is still poorly understood.

### 6.3. Infradian Rhythms and Therapy

One final biological cycle that we have recently learned plays a role in drug delivery to breast cancer is the estrus (or menstrual) cycle. The menstrual cycle is an infradian rhythm, a naturally occurring cycle in the human body that lasts longer than 24 h. In 2024, a Dutch group discovered that in three mouse models of breast cancer, the tumors displayed reduced responses to neo-adjuvant chemotherapy when treatment was initiated during the diestrus stage (roughly comparable to the luteal phase in humans), when compared with initiation during the estrus stage (comparable to the follicular phase and ovulation in humans) [124]. This finding is mechanistically plausible due to the systemic and localized changes that occur in the diestrus stage, such as a decreased tumor vessel diameter, making it more difficult to deliver drugs, and the elevated presence of macrophages patrolling for toxins and drugs, which could aid chemoresistance. The group originally aimed to explore this topic because the effectiveness of neo-adjuvant therapy is highly heterogeneous, even for tumors with the same molecular or pathological subtype. However, while humans do not have a distinct diestrus stage, similar findings were observed in retrospective premenopausal cohorts of human patients. These findings are enlightening because globally, over 30% of women diagnosed with breast cancer are premenopausal [125]. The physiology of the female body changes substantially during the menstrual cycle, but therapies have not been extensively adapted to or optimized for these oscillating physiological changes. This recent data warrants investigation in future clinical studies into the menstrual cycle as a factor that influences chemosensitivity of cancer cells to chemotherapy and, subsequently, the breast cancer outcome.

### 6.4. Artificial Intelligence and Computer-Aided Drug Design

In our new era of artificial intelligence (AI), we are seeing a deployment of new technologies and strategies that are aiding drug discoveries that target BCSCs. One of the most significant developments in CSC research is single-cell RNA sequencing, a technology that examines gene expression patterns of individual cells in a population. In one landmark publication in 2018 [126], researchers were able to divide human induced pluripotent stem cells (hiPSCs) into four different subpopulations based on their gene expression patterns: from most “stem-like,” to proliferative, to early primed for differentiation, and late primed for differentiation. These data can be applied to a machine learning algorithm, which is a type of computer program that learns patterns from data and makes predictions or decisions without being explicitly programmed for every possible scenario. The model can learn to classify each stem cell into one of four groups based on gene expression. Compared to existing methods, single-cell sequencing and a machine learning model improved classification accuracy and better identified cells transitioning between states.

Building upon prior research using single-cell RNA sequencing and machine learning, a recent publication from 2024 described the development of an AI program that screened for pre-existing compounds that induced differentiation in BCSCs [127]. Specifically, the program analyzed thousands of drug-induced gene expression profiles from a large public database (LINCS) to find drugs that might push BCSCs into differentiation. This process essentially turns the typically drug-resistant BCSCs into breast cancer cells that are more vulnerable to chemotherapy. By using an AI algorithm to discover these drugs, researchers can identify new uses for existing drugs without prior knowledge of their molecular mechanisms. This way, significant time and resources are saved, and the work that researchers need to do before moving into in vitro experiments is streamlined. To validate their findings, the researchers tested the candidate drugs on two breast cancer cell lines—MDA-MB-231 and MCF7—and found that five out of six successfully promoted BCSC differentiation and suppressed mammosphere formation. The drugs triptolide, OTS-167, quinacrine, granisetron, and A-443654 showed promise in treating breast cancer by specifically targeting BCSCs. Before the development of this AI model, attempts have been made to induce differentiation in BCSCs using well-established agents like retinoic acid [29]. However, these kinds of therapies have not gained a lot of traction, possibly because they are not specific to cancer stem cells and could affect normal stem cells too. Therefore, the hope is that technological innovations will help spur the development and discovery of agents to achieve targeted delivery. In the recent study using AI to select small molecules that induce differentiation, many hit molecules were drugs either already approved by the FDA or in clinical trials for other diseases. This underscores the amazing capability of AI technological advances to accelerate BCSC drug discovery.

Another future direction of BCSC drug development is using computer technology to create synthetic peptoids. Although traditional peptides are still a popular approach, as biologic compounds, they tend to have a short lifespan and instability that prevents them from being widely applicable against malignant tumors. Therefore, a new class of agents called peptidomimetics, or peptoids, has been introduced alongside recent advances in physical chemistry and biochemistry. Peptoids are peptidomimetics that contain N-substituted glycines, which provide improved proteolytic stability due to the absence of amide bonds in their backbone, and can be easily synthesized [128]. There is an enormous interest in applying peptoids to BCSC drug discovery. In 2021, Shukla et al. [129] discovered a peptoid ligand that specifically targets a structural protein, plectin, on lung CSCs. Their study found that their peptoid PCS2D1.2 significantly inhibited the NCI-H358 cell line (lung cancer) and prevented metastasis. Thus far, no research has been done that explores peptoids as inhibitors of BCSCs, so the field of peptidomimetics is yet another emerging field of BCSC research that remains largely unexplored.

## 7. Conclusions

BCSCs are responsible for the recurrence of breast cancer and its metastasis to distant sites. Protein markers, such as CD44, ALDH1, CD133, EpCAM, and CXCR1, exist that effectively identify and isolate BCSCs. However, there are significant barriers to translating investigational targeted therapies from preclinical success into effective clinical therapies due to tumor heterogeneity, the overlap of BCSC markers with normal stem cells, and BCSC inherent drug resistance. Recent advancements in immunotherapy and targeted inhibition of signaling pathways have generated some excitement. However, we still do not have FDA approval for any drug that explicitly targets BCSCs. Among the many challenges of targeting BCSCs, one of them is the off-target effects from investigational drugs harming normal tissue and normal stem cells. One novel putative strategy to avoid off-target effects is by employing a method such as dual-specific antibodies or innovative nanoparticles. We also addressed AI as an accelerator of drug discovery and chronotherapy to discover the temporal pattern of BCSC vulnerabilities, hoping that future advances toward more effective breast cancer treatments will prevent needless recurrences and tragic deaths.

## Figures and Tables

**Table 1:** Protein markers of BCSCs.

Marker	Description	Role in BCSCs	References
CD44	Cell-surface glycoprotein involved in cell adhesion and migration. EMT requires an isoform switch from CD44v to CD44s.	High CD44^+^/Low CD24^−^ phenotype marks BCSCs; linked to tumorigenicity and metastasis.	[4,18]
CD24	Surface protein containing sialic acid sugars that is involved in adhesion and co-stimulatory signaling to immune cells (like CD4^+^ T cells).	High CD44^+^/Low CD24^−^ indicates BCSCs with enhanced invasive capabilities, as opposed to the ALDH1^+^ BCSCs.	[4,18]
ALDH1 (endogenous marker)	Enzyme catalyzing oxidation of aldehydes and other important physiologic and toxicological functions.	High ALDH1 activity identifies BCSCs, especially the proliferative epithelial subtype associated with increased self-renewal and drug resistance.	[4,20]
CD133	Transmembrane glycoprotein associated with stemness as it suppresses differentiation, though its precise mechanism is unclear.	Marker for BCSCs with tumor-initiating capacity; overall, it has been associated with poor survival and prognosis.	[28]
EpCAM	An adhesion molecule located on the basolateral membrane of cells.	Helps BCSCs with invasion and metastasis, marker for isolating circulating tumor cells (CTCs) enriched with BCSC properties.	[4]
CXCR1	Chemokine receptor facilitating migration and invasion via the IL-8 axis.	Regulates BCSC activity; targeted by Reparixin in trials to reduce BCSC populations.	[26,29]
HER2	Receptor tyrosine kinase promoting cell growth and differentiation, and its downstream signaling pathway participates in crosstalk with other signaling pathways.	Overexpressed in HER2^+^ BCSCs; correlates with aggressive tumors by interacting with signaling pathways and increasing stemness features.	[4,30]
DCLK1	A CSC marker that is overexpressed in many types of cancer, including breast cancer	Overexpression of DCLK1 in tumor cells will likely contribute to stemness and self-renewal.	[31]
ABC Efflux Factors	CSCs express a high proportion of ABC transporters	The basis of a flow cytometry method to identify CSC	[32]

## Data Availability

The datasets and materials used and/or analyzed during the current study are available from the corresponding author upon reasonable request.
